# RNA Regulations and Functions Decoded by Transcriptome-wide RNA Structure Probing

**DOI:** 10.1016/j.gpb.2017.05.002

**Published:** 2017-10-12

**Authors:** Meiling Piao, Lei Sun, Qiangfeng Cliff Zhang

**Affiliations:** MOE Key Laboratory of Bioinformatics, Beijing Advanced Innovation Center for Structural Biology, Center for Synthetic and Systems Biology, Tsinghua-Peking Joint Center for Life Sciences, School of Life Sciences, Tsinghua University, Beijing 100084, China

**Keywords:** RNA structure probing, RNA structurome, RNA secondary structure, Structure–function relationship, RNA regulation

## Abstract

RNA folds into intricate structures that are crucial for its functions and regulations. To date, a multitude of approaches for probing structures of the whole transcriptome, *i.e*., **RNA structuromes**, have been developed. Applications of these approaches to different cell lines and tissues have generated a rich resource for the study of RNA **structure–function relationship**s at a systems biology level. In this review, we first introduce the designs of these methods and their applications to study different RNA structuromes. We emphasize their technological differences especially their unique advantages and caveats. We then summarize the structural insights in RNA functions and regulations obtained from the studies of RNA structuromes. And finally, we propose potential directions for future improvements and studies.

## Introduction

RNA is a molecule with diverse functions. In addition to transferring genetic information from DNA to protein, RNA can catalyze specific biochemical reactions, similar to the action of a protein enzyme. These RNA enzymes, *i.e.*, ribozymes, are vital to life by participating in a variety of basic biological processes, including RNA splicing, translation, and also tRNA biosynthesis [1]. Some RNAs, known as riboswitches, can also regulate gene expression by altering their own conformations in response to changes in the cellular environment or binding of ligands [2], [3], [4]. These, plus their ability to encode genetic information like many RNA viruses, have stimulated an interesting “RNA world” hypothesis, speculating that RNA may have been precursors to all life on Earth [5], [6].

One of the most significant findings in genomics in the last two decades is the discovery of pervasive transcription and the large number of non-coding RNAs (ncRNAs) in human transcriptome [7]. RNAs that have no or little coding potential and are longer than 200 nucleotides are collectively defined as long ncRNAs (lncRNAs) [8]. Many lncRNAs are found to carry out different types of functions, including regulating chromatin states and consequently gene expression, sponging small RNAs (sRNAs) and proteins for fast cellular regulations, or being scaffolds to bring together other RNAs and proteins to facilitate their cross-talking [8]. However, with the broad definition of lncRNAs and their big variations in sequence and expression, it is very challenging to understand what their functions are and how they are regulated.

Fortunately, there has been a well-established general rule that sequence determines structure determines function, especially for proteins [9]. RNA can also fold into intricate shapes by local and long-range pairing of nucleotides. Well-known examples include the aforementioned ribozymes, riboswitches, and some lncRNAs. Studies have shown that, like protein structures, RNA structures are critical for their correct functioning and aberrancy of RNA structures could possibly lead to human disease [10], [11]. It is thus a valid approach to study RNA functions and regulations from the perspective of RNA structures.

However, our current knowledge on RNA structures is very limited. Traditionally, structures of macromolecules are resolved with methods including X-ray crystallography, nuclear magnetic resonance (NMR) spectroscopy, and more recently cryo-electron microscopy (cryo-EM). Unfortunately, RNA molecules are usually much more flexible, dynamic, and thus structurally heterogeneous [12]. Therefore, it is usually very difficult to apply these techniques to obtain RNA structures. For example, X-ray crystallography requires that target structures form highly-ordered crystals. But in most cases, without the help of binding proteins, RNA molecules can adopt a big number of alternative structures, which makes crystallization challenging or even impossible. The large number of different conformations may also be well beyond the capability of new technologies like cryo-EM. Moreover, NMR spectroscopy is strongly limited to sRNAs and cannot be applied to study many other functional RNA molecules [13]. In addition, none of these methods can be used to study RNAs under cellular or physiological conditions.

Thus, for many years, our understanding on RNA structures and their functional relevance relies primarily on computational predictions. These predictions typically use thermodynamic calculation to obtain secondary structure models with lowest free energy [14], [15], [16], or sequence co-variation analysis to determine base-pairings that have been maintained through evolution [17], [18]. However, these approaches usually cannot take into account *trans*-acting factors like proteins, other RNAs, and small ligands, as well as other physiological conditions. As a consequence, they can only generate *in silico* secondary structural predictions of a given RNA alone. In addition, computational predictions do not work very well for big RNAs with complex structural elements like pseudoknots, kissing loops, or long-range interactions [19], [20].

Fortunately, recently we have witnessed the fast development of a new type of approaches, resurging from RNA structure probing analysis with chemicals and enzymes developed as early as 1970s [21]. It has been long known that a wide variety of chemicals and enzymes can react differently with RNA nucleotides in different structure context (Table 1). Many of these reactions leave footprints on the modified RNA molecules, which can be read out with gel filtration, or sequencing nowadays. Indeed, when combined with deep sequencing, these methods have the potential to reveal structures of the whole transcriptome, *i.e.*, RNA structurome, in a single experiment.

**Table 1:** Summary of high-throughput approaches to probing RNA structure

**Name**	**Type**	**Reagent**	***In vitro/in vivo***	**Organisms studied**	**Features and limitations**	**Refs.**
Enzymatic cleavage	PARS, PARTE	Nuclease S1 and RNase V1	*In vitro*	Yeast, human	F: PARTE can calculate RNA folding energies L: Nuclease cannot permeate through cell membrane, making *in vivo* study impossible; signal is rather sparse because of large size of enzymes	[22], [23], [24], [25]

	FragSeq	Nuclease P1	*In vitro*	Mouse	F: Similar to PARS, using samples without nuclease and with or without PNK as controls; focusing on short nuclear RNA to avoid fragmentation L: *In vitro* only; limited resolution	[26]

	ds/ssRNA-seq	RNase I, RNase V	*In vitro*	Arabidopsis, *Drosophila*, *C. elegans*	F: Sequencing the remaining regions after thorough digestion with ds/ssRNA nuclease L: *In vitro* only; limited resolution	[27], [28], [29], [30]

	PIP-seq	RNase one, RNase V1	*In vitro*	Arabidopsis	F: Revealing relationship between RBP occupancy and RNA secondary structure L: Assuming that removing RBP doesn’t affect RNA structure; *in vitro* only; limited resolution	[31]

Nucleotide modification	DMS-seq	DMS	*In vitro* and *in vivo*	Yeast, human	F: DMS can permeate through cell membrane, able to be used in living cells L: DMS has nucleotide bias, only able to react with adenines and cytosines	[32]

	DMS-MaPseq	DMS	*In vitro* and *in vivo*	Yeast, human	F: Utilizing the mutation rate caused by modification as the output signal instead of RT stop; higher signal-to-noise ratio L: Requiring higher sequencing depth; nucleotide bias	[33]

	Structure-seq	DMS	*In vitro* and *in vivo*	Plants	F: Similar to DMS-seq, including background control via detecting RT stops without DMS treatment L: Nucleotide bias	[34]

	Mod-seq	DMS	*In vivo*	Yeast	F: Similar to DMS-seq; focusing on rRNAs L: Nucleotide bias	[35]

	CIRS-seq	DMS, CMCT	*In vitro*	Mouse	F: Allowing to probe four nucleotides by combining DMS and CMCT; avoiding effects of RBP via deproteinization	[36]

	icSHAPE	NAI-N3	*In vitro* and *in vivo*	Mouse, human	F: The first *in vivo* SHAPE probing study; modified fragments further enriched by biotin isolation, making the signal super clean	[37]

	SHAPE-MaP	1M7, 1M6, NMIA		Synthetic RNA	F: Utilizing the mutation rate caused by modification as the output signal instead of RT stop; higher signal-to-noise ratio L: Requiring higher sequencing depth	[38]

	SHAPE-seq 1.0/2.0	1M7, NMIA, BzCN		Synthetic RNA	F: SHAPE has no bias toward four nucleotides L: SHAPE cannot permeate through cell membrane	[39], [40]

Base-pair crosslinking, proximity ligation	PARIS	AMT	*In vivo*	Mouse, human	F: Direct mapping of duplex groups; enrichment conducted by 2D gel filtration L: AMT can only crosslink uridine; efficiency of proximity ligation needs to be improved	[41]

	SPLASH	Biotinylated psoralen	*In vivo*	*E. coli*, yeast, human	F: Direct mapping of duplex group; enrichment conducted by biotin isolation L: Psoralen can only crosslink uridine; efficiency of proximity ligation needs to be improved	[42]

	LIGR-seq	AMT	*In vivo*	Human	F: Direct mapping of duplex group L: AMT can only crosslink uridine; efficiency of proximity ligation needs to be improved	[43]

*Note*: PARS, parallel analysis of RNA structures; PARTE, parallel analysis of RNA structures with temperature elevation; FragSeq, fragmentation sequencing; ds/ssRNA-Seq, double-stranded RNA-Seq and single-stranded RNA-Seq; PIP-seq, protein interaction profile sequencing; DMS-MaPseq, dimethyl sulfate mutational profiling with sequencing; Mod-seq, map RNA chemical modification using high-throughput sequencing; CIRS-seq, chemical inference of RNA structures followed by massive parallel sequencing; SHAPE, selective 2′ hydroxyl acylation analyzed by primer extension; icSHAPE, *in vivo* click SHAPE; SHAPE-MaP, SHAPE and mutational profiling; PARIS, psoralen analysis of RNA interactions and structures; SPLASH, sequencing of psoralen crosslinked, ligated, and selected hybrids; LIGR-seq, ligation of interacting RNA and high-throughput sequencing; DMS, dimethyl sulfide; CMCT, 1-cyclohexyl-(2-morpholinoethyl)carbodiimide metho-p-toluene sulfonate; NAI-N3, 2-methylnicotinic acid imidazolide-azide; 1M7, 1-methyl-7-nitroisatoic anhydride; 1M6, 1-methyl-6-nitroisatoic anhydride; NMIA, N-methylisatoic anhydride; BzCN, benzoyl cyanide; AMT, 4′-aminomethyl trioxsalen; PNK, polynucleotide kinase; RBP, RNA binding protein.

In this review, we will introduce the designs of these methods and their applications to study RNA structuromes in different species. We will emphasize their technological differences, especially their unique advantages and caveats. We will then summarize the structural insights into RNA functions and regulations obtained from the studies of RNA structuromes. And finally, we propose potential directions for future improvements and studies.

## Methods for probing RNA structurome

Different enzymatic cleavages and chemical modifications have distinct preferences toward single-stranded RNA (ssRNA) and double-stranded RNA (dsRNA). RNA structure probing approaches are designed to utilize these structural preferences. The technological development follows a path from low-throughput to genome-wide, from *in vitro* to *in vivo*, and from one-dimension to two-dimension analyses (Table 1).

### Enzymatic cleavage methods

Endonucleases were found to have structural specificity decades ago [44]. Since then, a variety of nucleases have been used to generate cleavage sites that contain structural information. For instance, RNase A and T1 cleave unpaired regions and generate products with 5′-OH and 3′-P end [45], [46], whereas RNase V1 only cleaves paired regions and generates 3′-OH and 5′-P end [47].

**Figure 1:**
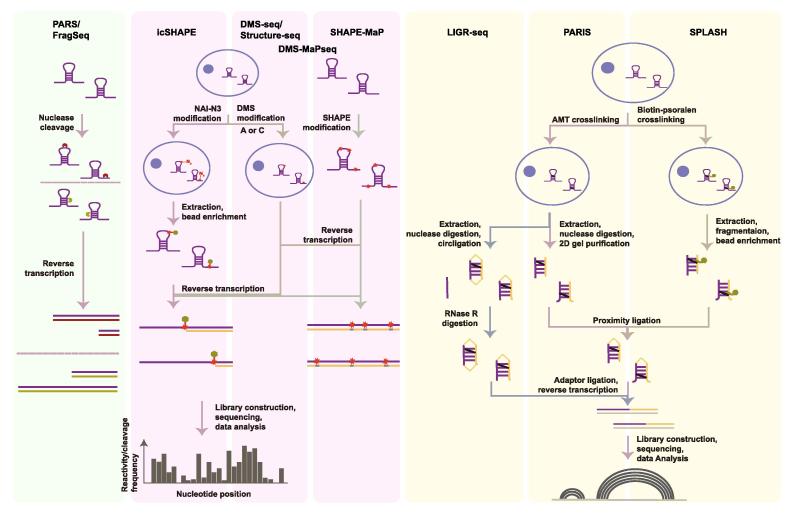
**Experimental workflow of some representative high-throughput approaches for RNA structure probing** PARS, parallel analysis of RNA structures; FragSeq, fragmentation sequencing; SHAPE, selective 2′ hydroxyl acylation analyzed by primer extension; icSHAPE, *in vivo* click SHAPE; SHAPE-MaP, SHAPE and mutational profiling; DMS-MaPseq, dimethyl sulfate mutational profiling with sequencing; LIGR-seq, ligation of interacting RNA and high-throughput sequencing; PARIS, psoralen Analysis of RNA interactions and structures; SPLASH, sequencing of psoralen crosslinked, ligated, and selected hybrids; DMS, dimethyl sulfide; NAI-N3, 2-methylnicotinic acid imidazolide-azide; AMT, 4′-aminomethyl trioxsalen.

Nuclease digestion is then followed by high-throughput sequencing to read cleavage sites (Figure 1). Parallel analysis of RNA structures (PARS) is the first study to obtain mRNA secondary structure profile at transcriptome level, by utilizing RNase S1 and RNase V1 to cut ssRNA and dsRNA regions, respectively [22]. The ratio of cleavage sites of RNase V1 to RNase S1 is calculated as a PARS score to represent the tendency to form RNA secondary structures at single-nucleotide resolution. Similarly, parallel analysis of RNA structures with temperature elevation (PARTE) applies RNase-seq of V1 at a series of elevating temperatures to calculate RNA folding energies [23], whereas fragmentation sequencing (FragSeq) relies on nuclease P1 to cleave ssRNA [26]. These methods all focus on analysis of cleavage sites as output from the experiments. Alternatively, dsRNA-Seq and ssRNA-Seq (ds/ssRNA-Seq) look for enriched RNase-insensitive ssRNA and dsRNA regions, respectively, after thorough digestion by dsRNase and ssRNase [27], [28], [29]. Protein interaction profile sequencing (PIP-seq) incorporates ds/ssRNA-Seq with crosslinking methods. For PIP-seq, RNAs are firstly crosslinked with proteins and ds/ssRNA-Seq is applied with and without proteinase treatment [31]. It is also noteworthy to mention that hydroxyl radical can react with RNA riboses exposed to the solvent and lead to RNA cleavage. Hydroxyl radical footprinting (HRF) profiles RNA solvent-accessibility by sequencing these RNA cleavage sites [48].

Using the enzymatic cleavage methods, *in vitro* RNA structuromes of multiple species have been successfully generated. In particular, deproteinized PARS is able to measure near *in vivo* RNA structures [22]. The main drawback of these methods, however, is that normally nucleases are not permeable through the cell membrane, making *in vivo* probing very challenging or impossible.

### Nucleotide modification methods

Some chemicals can modify RNA nucleotides in specific structure contexts. Some of these modifications can block reverse transcription (RT) preceding the modified sites and thus allow for detection by reading RT stop sites. Nucleotide modifications mainly fall into two groups: base modification and backbone modification. Dimethyl sulfide (DMS) is a base modification reagent, which is frequently used to alkylate Watson–Crick (WC) face of unpaired adenine (A) and cytosine (C), while 1-cyclohexyl-3-(2-morpholinoethyl) carbodiimide metho-p-toluene sulfonate (CMCT) reacts with unpaired uracil and guanine (G) [49], [50]. Different experiments have been designed based on DMS modification (Figure 1). DMS-seq probes enriched DMS modifications *in vivo*, *in vitro*, or after RNA denaturation [32]. Structure-seq also includes a background control without DMS treatment, which properly excludes natural RT stops [34]. Mod-seq is similar but assesses secondary structure of rRNA instead of enriched mRNA [35]. The recently-developed DMS-MaPseq method uses reverse transcriptase mismatch rather than truncation products in collecting RNA structure information, thus improving signal-to-noise ratio [33]. One of the main limitations of DMS, however, is that it only provides profiles of A and C. Chemical inference of RNA structures followed by massive parallel sequencing (CIRS-seq) thus combines DMS and CMCT to cover all four nucleobases in natively-folded RNA with deproteinization [36].

RNA base-pairing brings geometric constraints to ribose involved in secondary structure, and thus protects the backbone from chemical modification [51]. Selective 2′ hydroxyl acylation analyzed by primer extension (SHAPE) is a method based on backbone modification for probing RNA secondary structure [51]. SHAPE reagents, like 1-methly-7-nitro-isatoic anhydride (1M7) and *N*-methylisotoic anhydride (NMIA), specifically modify the ribose of unstructured nucleotides without any bias toward one or more of the four nucleobases. Notably, complex structures besides canonical base pairing are also detectable by SHAPE reagents, allowing for probing more complicated interactions. SHAPE-seq was designed by adapting SHAPE methods in combination with high-throughput sequencing [52]. Although limited by permeability of the probing reagents (1M7 and NMIA), SHAPE-seq provides the unbiased RNA secondary structure profiling. SHAPE modification has been found to generate mutations during RT reaction after alteration of RT reaction conditions [38]. Based on this discovery, SHAPE-MaP locates the modification sites by analyzing mutation sites [38] (Figure 1). SHAPE reagents were initially used to probe RNA structure *in vitro*. But cell-permeable ones, *e.g.*, 2-methyl-3-furoic acid imidazolide (FAI) and 2-methylnicotinic acid imidazolide (NAI), can be used for *in vivo* RNA structure probing [53]. A newly-developed technology, *in vivo* click SHAPE (icSHAPE) uses an optimized SHAPE compound NAI-N3 with increased permeability and incorporates biotin-streptavidin isolation to enrich modified RNA fragments [37] (Figure 1). The biotin-streptavidin isolation system is introduced to the clickable azide moiety of icSHAPE reagent, thereby achieving higher sensitivity for modified RNAs.

### Cross-linking methods

The two aforementioned types of methods only focus on detecting which regions of RNA are single-stranded and which are double-stranded. Information on another big part of RNA structure, the detailed intermolecular or intramolecular base-pairing pattern, however, is missing. Psoralens are well known mutagens that crosslink DNA or RNA duplexes by forming adducts with adjacent thymines (Ts) or uridines (Us) when activated by UV photon [54], [55]. Three recent studies, psoralen analysis of RNA interactions and structures (PARIS), sequencing of psoralen crosslinked, ligated, and selected hybrids (SPLASH), ligation of interacting RNA and high-throughput sequencing (LIGR-seq), utilize psoralens to crosslink the duplex regions of RNA [41], [42], [43] (Figure 1). The RNA is then fragmented and retrieved after RNase and protease digestion. Ends of the crosslinked fragment duplex are then ligated via proximity ligation, followed by reverse crosslinking and library construction for sequencing. After mapping, gapped reads are collected as indication of direct base-pairing.

Apparently, proximity ligation cannot tell duplex regions from unpaired fragments. Various methods are thus used in order to enrich duplex regions to reduce background noise. PARIS involves 2D gel electrophoresis to separate duplexes from unpaired regions, which gets clean duplexes but lowers the yield at the same time [41]. SPLASH uses biotinylated psoralen as the crosslinking reagent, which makes it possible to enrich duplexes by streptavidin beads [42]. LIGR-seq employs RNase R to digest uncircularized RNA after proximity ligation by CircLigase [43]. RNase R digestion, however, is performed after ligation, since ssRNAs still can be ligated and confound the results at this stage.

## Insights of RNA functions and regulations from RNA structurome

RNA structure is crucial for gene function and regulation by influencing RNA transcription, processing, localization, translation, and degradation. The canonical roles of RNA structure in many different biological processes have been reviewed elsewhere [56], [57], [58], [59], [60], [61]. Thanks to the transcriptome-wide RNA structure probing, we are now able to understand how RNA structure is regulated and functions at a systems level. Here, we only briefly summarize the novel insights into functional significance of RNA structure in various cellular processes from these systems biology studies.

### Transcription

The life cycle of a RNA molecule begins with its transcription. There is accumulating evidence showing that RNA folds into structures along with its transcription, and this co-transcriptional folding plays a critical role in defining RNA functions [62], [63], [64], [65], [66]. Intermediate structures often form and present for a certain period of time when transcription proceeds from the 5′ to the 3′ end of the RNA sequence [67], [68], and later concess to globally more stable conformations [63], [69].

The relationship of RNA transcription and those transient intermediate structures remains elusive for most cases. A study in 1980s on the *tryptophan* (*trp*) operon showed that the formation of transient RNA structures is influenced by the interplay of co-transcriptional RNA folding and translation [70]. The leader of this *trp* operon encodes a tryptophan-rich peptide. Its transcript can assume two alternative structural elements: the attenuator and the anti-terminator. While the attenuator structure prevents further transcription, the anti-terminator permits it. These two structure elements form co-transcriptionally and are regulated by the binding and activity of ribosomes to the leader.

We currently do not have much insight into the *in vivo* RNA folding pathways during transcription. None of the aforementioned high-throughput probing experiments has investigated the interplay between RNA structures and transcription. However, combining nascent RNA sequencing with structure probing, or using other methods to carefully isolate different stages of transcripts, we may be able to study their intricate relationships. In addition, recent progresses on *in vivo* methodology development are likely to improve our ability to interrogate the role of RNA structures in transcription, and *vice versa* [71], [72].

### Processing

Most nascent RNAs are subjected to further processing, including capping, splicing, and polyadenylation before they are better prepared to meet their functional roles. Among those, alternative splicing (AS) is a widespread means that vastly increases transcript and protein diversity [73], [74]. RNA splicing involves many *cis*-acting sequence elements. The basic ones are important for spliceosome binding and splicing reaction, including the 5′ splice site, the branch-point, and the 3′ splice site. In addition, several classes of auxiliary regulatory signals that play critical roles in splicing regulation have been defined as well. These include exon splicing enhancers (ESEs) and silencers (ESSs), as well as intron splicing enhancers (ISEs) and silencers (ISSs), categorized based on their location and their effects on splicing. Splicing factors that recognize and bind to the enhancer/silencer elements are defined as activator/repressor proteins, respectively. The structural effects of RNA on splicing involve these basic and auxiliary elements, as well as their interplay with the spliceosome complex and splicing factors.

Many examples have shown that RNA structure can regulate AS, by affecting spliceosome recognition of basic *cis*-acting elements [75] or through influencing splicing factor binding to auxiliary elements [76]. There also exists another type of RNA structure regulation on splicing, *i.e.*, forming (usually long-range) base-pairings that facilitate joining of a common exon to different alternative exons. An incredibly interesting example comes from the AS of the *Drosophila* gene encoding Down syndrome cell adhesion molecule (*Dscam*) [77]. There have been some nice reviews that summarize individual instances in all these three aspects [58], [78], [79], [80]. Here we only focus on general structural observations of splicing obtained in recent high-throughput structure probing experiments.

**Figure 2:**
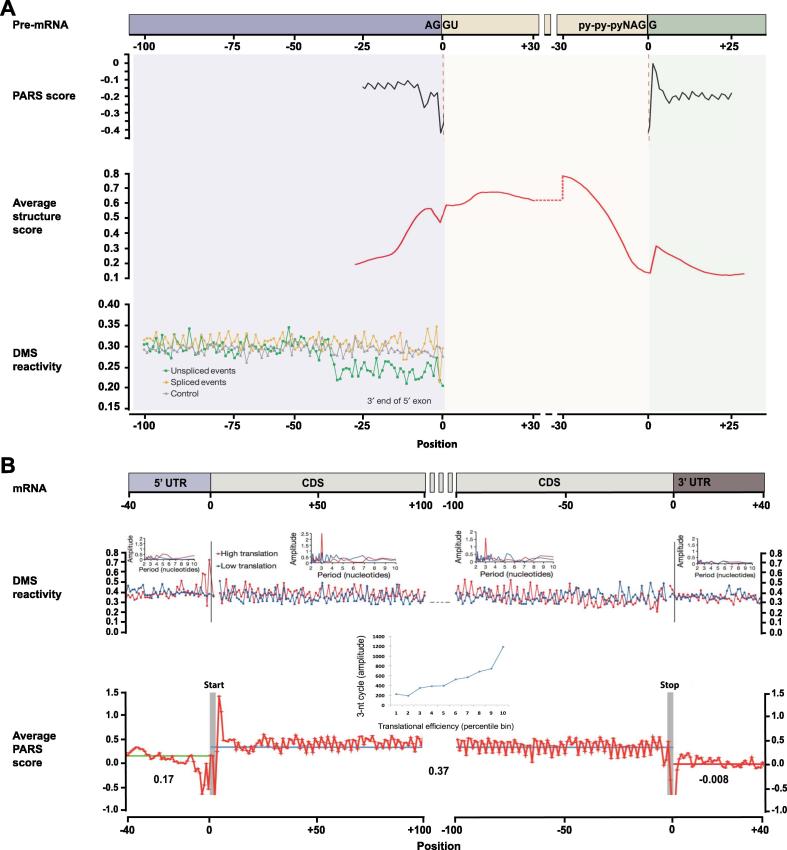
**Structural landscapes of RNA splicing and translation** Structural landscapes of RNA splicing and translation are shown in panels **A** and **B**, respectively. Note that difference structural score metrics are used in different technologies. For PARS and structure scores, the higher score means the more secondary structures, while for DMS reactivity, the higher the score means the fewer secondary structures. The figure is adapted from [25], [36], [39], [74]. PARS: parallel analysis of RNA structures; DMS: dimethyl sulfide.

RNA structure can regulate splicing by directly interfering with spliceosome binding sites. In the PARS study of the *in vitro* human structurome, RNA structure signals were screened across exon-exon junctions in already spliced mRNAs. It is observed that the splicing donor dinucleotides AG are more accessible compared to nearby nucleotides, whereas the acceptor nucleotide G/A tends to be more structured [24] (Figure 2). Further analysis of the structural signal of pre-mRNA splicing in *Arabidopsis* structurome using ds/ssRNA-Seq confirmed this signature on splicing donor/acceptor sites with more details in flanking intronic regions [30]. The structure score of splicing donor, however, is higher than that of splicing acceptor in *Arabidopsis* structurome, which is opposite in human structurome. In a later study of the *in vivo Arabidopsis* structurome, Ding et al. revisited RNA splicing using the Structure-seq. They found that in the region upstream of the splicing donor site, structures are less accessible for the unspliced events than for the spliced events [34]. This suggests that secondary structure at the splice donor sites may disfavor splicing. This finding is consistent with the ds/ssRNA-Seq study of *in vivo Arabidopsis* structurome for U12-type introns and constitutive introns [30].

RNA structures can also regulate splicing by directly affecting splicing factor binding. The icSHAPE study of the mouse structurome analyzed RNA structure signatures in auxiliary regulatory sites [37], focusing on the splicing factor Rbfox2 (fox-1 homolog in mouse), a member of the “feminizing locus on X” (Fox) family of RNA-binding proteins (RBPs) [37]. Spitale and colleagues compared *in vivo* and *in vitro* RNA structures at Rbfox2-binding sites and found high level of differences, suggesting a strong structural effect of the splicing factor binding *in vivo*. The structural signatures and rearrangement were later shown to be effective in identifying true Rbfox2-binding sites [37]. The structure significance in defining a true splicing factor binding sites was also exemplified in a later study on the binding of heterogeneous nuclear ribonucleoprotein C (hnRNPC) to polyU tracts [81]. By integrative data analysis of m^6^A modification, RNA structures, and RBP binding sites [82], Hafner et al. found that m^6^A in the complementary strands of U-rich hairpins weakens the hairpin secondary structure and promotes hnRNPC binding. In addition, knockdown of two genes encoding m^6^A methyltransferases, *METTL3* and *METTL14*, reduced hnRNPC binding and affected AS through disrupting hairpin structures [81].

### Localization

The majority of RNAs are localized to distinct cellular domains with exquisite temporal and spatial control, providing an important mechanism for gene expression regulation [59], [83], [84]. RNA localization is usually controlled through a set of *cis*-acting elements present in the RNA, which encode the cellular “address” of the host transcripts. These *cis*-acting elements, primarily located within the 3′ UTR, are called “localization elements” or “zipcodes” [85]. And a combination of RBPs, which often function in association with cytoskeletal motors, recognize these zipcodes to regulate RNA transport throughout the cell [86], [87].

Accumulating evidences suggest that not only the sequences but also the structures of these zipcodes are critical for RNA localization. For example, a study showed that a stem-loop structural element, BLE1, is critical for the transport of the host *bicoid* mRNA from the nurse cells into the oocyte [88]. More interestingly, sometimes the primary sequences lack conserved sequence zipcodes [89]. Therefore, efforts have been concentrated on the discovery of conserved structural motifs of zip codes, in particular stem loops [90], [91].

The experimental studies of RNA structures can potentially shed light on zipcode discovery. The analysis of the *Saccharomyces cerevisiae* structurome has revealed that mRNA encoding proteins with specific sub-cellular locations or involved in some metabolic pathways are more structured in the coding region [22]. On the contrary, mRNAs that encode subunits of the ribosome tend to have much less structure in their 5′ UTR and coding sequences. More structural analyses, however, are needed to scrutinize, identify, and annotate structural motifs from this rich set of data.

RNA of some secretory proteins is exported from the nucleus by using a signal sequence coding region (SSCR) in the transcripts [92]. In the aforementioned study of the *S. cerevisiae* structurome, Kertesz et al. examined the structures of the transcripts that are predicted to encode a signal peptide. They found that the SSCRs and their proximity sequences have a lower PARS score, suggesting that specific secondary structures may assist RNA nuclear export [22].

### Translation

Many RNAs are made for translation. Long before the high-throughput probing experiments, it had been observed that RNA structure plays an important role in translation regulation. For example, the temperature-sensitive structures, *e.g.*, RNA thermometers or riboswitches, are able to affect mRNA translation [93]. These RNA structures adopt different conformations to inhibit or allow the binding of ribosomes, thus regulating expression of the encoded proteins [94]. Another example is from a study in 1980s. It was found that structures formed around the translation start site of an mRNA impede its translation initiation, a rate-limiting step that significantly influences translation efficiency [95]. This finding was later confirmed by a large-scale study calculating the mRNA folding near the ribosomal binding site and for correlation with protein abundance [96]. A later transcriptome-wide study repeated this by correlating predicted RNA structure [97] with translation efficiency from experimental polysome profiling [98]. In addition, using computational analysis, Shabalina et al. predicted an interesting distinguishing feature of the CDS region, *i.e.*, a three-nucleotide periodicity in mRNA secondary structure [99]. This intriguing feature was later confirmed by many large-scale *in vitro* and *in vivo* RNA structure probing experiments [22], [32], [34], [37]. The structural landscape of translation elements, including those of 5′ UTR, start site, CDS, stop site, and 3′ UTR, however, is much more complex as later revealed in these transcriptome-wide experimental studies. We will summarize relevant findings below.

The first whole-genome structural probing experiment was performed on HIV-1 genomic RNA using SHAPE technologies, focused on structure–translation relationships [100]. It was found that both the 5′ UTR and the 3′ UTR are associated with increased level of RNA secondary structures than coding region. Interestingly, there exists a distinct pattern of structures in coding region, which correlates well with protein and domain boundaries. These findings implicate a role of RNA structure in translation pausing and co-translational protein folding.

However, following studies of PARS [22], [24], ds/ssRNA-seq [28], [29], DMS-seq [32], and Structure-seq [34] experiments performed on different organisms revealed that the relative structural contents vary among 5′ UTRs, 3′ UTRs, and the coding regions (Figure 2). The studies on Drosophila, *Caenorhabditis elegans*, and human mRNAs agreed with the HIV-1 analysis, whereas opposite results were obtained for yeast and *Arabidopsis*. It is not entirely clear whether this is due to *in vitro* or *in vivo* structure features probed by different technologies or characteristics defined by species.

Nevertheless, the low structure contents around the start and stop codon, and also the three-nucleotide periodicity have been universally observed [22], [24], [34], [36] (Figure 2). These studies further painted a finer global view of structure–translation relationships. In a study examining RNA structures in *S. cerevisiae* using PARS, Kertesz et al. observed that mRNA structure of the translation start site are negatively correlated with ribosome density throughout the transcript [22]. Notably, they also found that the three-nucleotide periodic repeat pattern is significantly correlated with translation efficiency. Given the *in vitro* nature of the aforementioned study, this observation was later revisited and confirmed in the *Arabidopsis* structurome study *in vivo* [34].

Moreover, an interesting positive correlation between the level of mRNA structure and its overall ribosome association has been revealed in another study of *Arabidopsis* structurome using ds/ssRNA-seq [29]. It is possible that mRNA structure could slow down or even stall the translocation of ribosomes and cause them to form clusters on mRNAs. Further investigation is needed to examine whether the increased ribosome association would affect protein translation and consequently its abundance.

The translation rates are not uniform along the CDS region. *In vitro* studies have suggested that the presence of RNA secondary structure promotes ribosome pausing [101]. However, complicated by multiple factors involved, including RNA structure, tRNA abundance, and codon choice, it is difficult to figure out how RNA structures *in vivo* may influence ribosome pausing, begging for more integrated quantitative studies. Interestingly, a mouse embryonic stem (ES) cell structurome study using icSHAPE technology [37] revealed a distinctive structure signature at ribosome pause sites: more structures at the exit (E) and peptidyl-tRNA (P) sites, and fewer structures at the aminoacyl-tRNA (A) site. This structural pattern was also observed *in vitro* when ribosome binding to mRNA is depleted, and in negative control sites with similar sequence context. This suggests that the RNA structure pattern of ribosome pausing sites is probably encoded in their sequences. Notably, the flanking 5′ region of the negative control sites showed the lack of typical three-nucleotide periodic signal, suggesting it may play some role in ribosome pausing regulation [37].

Recently, a structurome study of yeast and human using psoralen crosslinking reported again that dense structures around the start codon could inhibit RNA translation, whereas structures of long-range 5′-to-3′ interactions could promote translation [42]. Interestingly, it was found that large RNA conformational changes *in vivo* could change translation efficiency, suggesting a potential mechanism for translation regulation. In summary, all these genome-wide studies show that mRNA structures exert a significant effect on its translation at multiple levels in various organisms.

### Stability and degradation

RNA is degraded in a carefully-controlled way at the end of its life cycle. RNA structures are also found to be involved in RNA stability and degradation as well. For instance, in addition to regulating translation, the small structural elements of riboswitches can also influence RNA stability [102]. In eukaryotes, RNA degradation is mainly accounted for by the exosome complex, an exonuclease that works from the 3′ to the 5′ end and needs an ssRNA region of about 30-nucleotide in the 3′ end of its targets [103]. This is consistent with the recent crosslinking study of yeast and human structuromes, which suggest that RNAs with structures present in the 5′ end only are associated with faster degradation, whereas structures at the 3′ end could inhibit exosome-mediated RNA decay [42].

It is then natural to speculate that RNA stability is positively correlated with RNA structure content in its 3′ end. In a study on *in vitro* structurome of *S. cerevisiae*, the RNA folding energies were measured using PARTE, an expansion of the PARS technology by applying it at different temperatures [23]. As a result, it was found that mRNAs with low average melting temperatures (*i.e.*, less structured) decreased most rapidly in abundance following heat shock. Notably, inactivation of the exosome significantly decreased the degradation of mRNAs with unstable structures.

This finding is consistent with a study probing *E. coli.* structurome *in vitro* using ds/ssRNA-seq. Del Campo et al. revealed that mRNA abundances are positively correlated with CDS secondary structures [25]. Conversely, mRNA abundances are found to be significantly negatively correlated with RNA structures in *Arabidopsis* [29]. Using Degradome sequencing, a weak but significant positive correlation was detected between mRNA structure and its turnover, suggesting that mRNAs of high level of structures are associated with increased rate of degradation. Further examination with small RNA-Seq analysis revealed a strong positive correlation between mRNA structure and sRNA abundance. This raises an interesting hypothesis that that RNA structures may be cleaved and processed into sRNAs [29].

## Concluding remarks and outlook

Transcriptome-wide RNA structure maps, *i.e.*, RNA structuromes, and studies of structure–function relationships have generated many new insights into RNA biogenesis, processing, localization, translation, and degradation. To date, studies have been focusing on general principles of the most basic biological processes. In the future, more in-depth investigations are needed for specific biological events in certain cell lines, tissues, environments, or conditions including human diseases. For example, what are the roles of structure in the infection of RNA virus? Is RNA structure an important regulator in early development when transcription is silent? Can we find any RNA structure biomarkers in human disease and could they be a diagnosis target for disease development? Comparative studies are especially required to uncover structures that may be causative factors or direct effectors. The future of these applications is to infer RNA functions based on data mining and classification of structure elements in different biological contexts, thus providing a knowledgebase for functional and mechanistic studies.

In addition to high quality data generation, well-designed bioinformatics analysis is the key to these applications. Technologies have been developed including a computational framework that processes sequencing data. The data processing follows normal RNA-seq analysis pipelines that include sequencing data quality control and trimming, reads alignment and abundance estimation. It also calculates a structure score to represent the preference for individual nucleotide or a sequence region to be single or double-strand. For enzymatic cleavage and nucleotide modification technologies, the structure score of a nucleotide is normally defined as the ratio of the number of RT stops or cleavage sites mapped to that position divided by the same number in a control experiment, in normal or log space [22], [24], [34], [37], [39], [40]. More accurate algorithms with sophisticated statistical models are developed later for some methods. For example, Ouyang et al. used hypergeometric tests with false discovery rate adjustment to identify reliable structural states and then incorporate the information for RNA structure inference [104]. Aviran et al. introduced probabilistic framework that models polymerase drop-off and chemical modification, and uses maximum-likelihood estimation to infer structural state for every nucleotide [105]. Zou and Ouyang developed a joint Poisson-gamma mixture to model multiple RNase-seq data and combine it with hidden Markov model to infer RNA structures [106]. In a recent study, a beta-uniform mixture hidden Markov model is used to calculate a statistically interpretable score for nucleotide structure preference [107]. These new methods usually can yield higher accuracy for RNA structure inference or generate confident structure estimations at much lower sequence coverage levels.

Better structure probing technologies that combine the strengths of new chemicals and creative sequencing designs are also desired to provide more accurate and comprehensive structure information. For example, most probing methods, except for the SHAPE-MaP [38] and the recently-developed DMS-MaPseq [33], use reverse transcriptase truncation products to collect RNA structure information. However, as shown in the analysis of DMS-MaPseq, using reverse transcriptase mismatch can improve signal-to-noise ratio and can be used to probe structures of low-abundant transcripts. More importantly, the ability to report multiple structure features per sequencing read allows for probing RNAs in multiple conformations and single-molecule structure analyses based on co-occurrence of DMS modifications on one read.

Most probing methods that use enzymatic cleavage or base and sugar modifications only generate one-dimensional averaged structure information. Newly-developed methods that use UV or psoralen crosslinking can provide two-dimensional information, but their resolution and coverage are so far limited (*e.g.*, PARIS, SPLASH, and LIGR-seq [41], [42], [43]). Tools that can greatly improve our ability to obtain direct base-pairing information include: (a) crosslinkers that can connect different bases with high efficiency; (b) methods that can locate the exact sites of base-pairing; and (c) computational pipelines with higher resolution and accurate duplex confidence calculation.

It has been a long-standing question on the interplay between RNA structure and RBP binding. It is also of great interest to find out how this interplay would affect RNA probing. The PIP-seq method is able to obtain information on both protein binding and RNA structures. Silverman et al. analyzing RBP-binding sites in *Arabidopsis* and found that most of these sites were more of single-stranded fragments flanked by structured regions [31]. However, it remains a big myth whether the structure signature is a cause or a consequence. It is possible both could be true as the interplay of protein binding and RNA structures is complex and possibly varies from one protein to another. With the rich resource of RNA structurome data and RBP binding data from high-throughput CLIP experiments [108], [109], it is now becoming possible to carry out a systematic study to investigate the relationship between RNA structure and RBP binding. But it should be noted that some structure probing methods could possibly generate biased information for this types of analysis. For example, RBP binding could cause steric effects that lead to inefficient cleavage or modification [110], [111].

A long-term goal of the RNA structure study is to construct structure models of RNA and protein (RNP) complexes. To date, very limited number of RNA 3D experimental structures are available in the PDB database [112]. In the future, it would be necessary to integrate secondary structure probing methods with 3D methods like crystallization, NMR, and in particular cryo-EM, small angle X-ray scattering (SAXS) technologies. Although the development in cryo-EM now allows for structure determination of RNP complexes with near atomic resolution [113], [114], [115], [116], it remains very challenging and success has been limited to a few cases. Nonetheless, cryo-EM, as well as SAXS, is very efficient in capturing overall shapes of big RNP complexes. Secondary structure probing should be able to help identify stable RNA structural domains to be fitted into the shape of the whole big RNP complexes. Finally, computational modeling will generate a high-resolution and complete picture of RNP complexes to help elucidate their regulations and functions and shed light on the mechanism and treatment of diseases related to RNA structures.

## Competing interests

None.
